# The human organic cation transporter OCT1 mediates high affinity uptake of the anticancer drug daunorubicin

**DOI:** 10.1038/srep20508

**Published:** 2016-02-10

**Authors:** Emil Andreev, Nicolas Brosseau, Euridice Carmona, Anne-Marie Mes-Masson, Dindial Ramotar

**Affiliations:** 1 Maisonneuve-Rosemont Hospital, Research Center, Université de Montréal, Department of Medicine, 5415 Boul. de l’ Assomption, Montréal, Québec, Canada, H1T 2M4; 2 Institut du cancer de Montréal/Centre de recherche du Centre hospitalier de l’Université de Montréal (CRCHUM), 900 rue Saint-Denis, Montréal, Québec, Canada, H2X 0A9; 3 Department of Medicine, Université de Montréal, 2900 Boulevard Edouard-Montpetit, Montréal, Québec, Canada, H3T 1J4

## Abstract

Anthracyclines such as daunorubicin are anticancer agents that are transported into cells, and exert cytotoxicity by blocking DNA metabolism. Although there is evidence for active uptake of anthracyclines into cells, the specific transporter involved in this process has not been identified. Using the high-grade serous ovarian cancer cell line TOV2223G, we show that OCT1 mediated the high affinity (*K*_*m*_ ~ 5 μM) uptake of daunorubicin into the cells, and that micromolar amounts of choline completely abolished the drug entry. OCT1 downregulation by shRNA impaired daunorubicin uptake into the TOV2223G cells, and these cells were significantly more resistant to the drug in comparison to the control shRNA. Transfection of HEK293T cells, which accommodated the ectopic expression of OCT1, with a plasmid expressing OCT1-EYFP showed that the transporter was predominantly localized to the plasma membrane. These transfected cells exhibited an increase in the uptake of daunorubicin in comparison to control cells transfected with an empty EYFP vector. Furthermore, a variant of OCT1, OCT1-D474C-EYFP, failed to enhance daunorubicin uptake. This is the first report demonstrating that human OCT1 is involved in the high affinity transport of anthracyclines. We postulate that OCT1 defects may contribute to the resistance of cancer cells treated with anthracyclines.

Members of the anthracycline family such as doxorubicin (DOX) and daunorubicin (DNR) are hydrophilic anticancer agents that are transported into cells where they intercalate into the DNA, disrupt the function of DNA polymerase, and promote cell death^1^. Anthracyclines exhibit high tissue-penetration and retention in nucleated cells, and are used for treating a variety of cancers that include leukemia and lymphomas, as well as breast, lung, ovarian, gastric and thyroid cancers^2^. However, a significant fraction of these cancer types is resistant to anthracyclines. Notably, over 50% of older patients (>60 yrs) with acute myeloid leukemia (AML) do not achieve complete remission after induction chemotherapy with DNR, and in some cases, remission is short-lived due to drug resistance^3^,^4^,^5^.

A characteristic mechanism associated with drug resistance is the elevated levels of plasma membrane ABC transporters^6^. These include the multidrug resistant efflux pump, MDR1/ABCB1, and the multidrug resistant-associated protein, MRP1, which are known to increase efflux of chemotherapeutic agents, allowing tumor (and normal) cells to evade drug-induced cytotoxicity^6^,^7^. Drug efflux transporters are known to be upregulated in some AML patients, and there is evidence suggesting that ABCB1 can expel DNR from the cells^8^. However, inhibition of ABCB1 with valspodar did not improve outcomes for/treatment in drug-resistant AML patients^8^. This suggests that other alternative mechanisms are involved in anthracycline resistance. These mechanisms are likely to involve (i) defects in DNR entry into cancer cells via uptake transporter(s), (ii) enhanced sequestration of DNR by lysosomes for detoxification and (iii) increased repair of DNR-induced DNA lesions. To date, the uptake transporter allowing entry of DNR into cells has not been identified^9^. Such a transporter could represent a critical pathway that, when defective, leads to drug refractory disease.

Human cells possess three high affinity (OCTN1/SLC22A4, OCTN2/SLC22A5 and hCT2/SLC22A16), as well as three low affinity (OCT1/SLC22A1, OCT2/SLC22A2 and OCT3/SLC22A3) L-carnitine transporters^10^. These are versatile organic cation transporters that possess varying affinities for a range of substrates, including several anticancer drugs such as bleomycin and oxaliplatin^11^,^12^. Okabe *et al*. (2005) initially reported that hCT2 has the ability to transport DOX, although the evidence remains circumstantial^9^. In their study, the authors injected hCT2 cRNA into the oocytes of *Xenopus laevis* and determined a high affinity for the uptake of DOX with an apparent *K*_*m*_ of 5.2 μM^9^. However, the characterization of hCT2 as a DOX-transporter was limited as competition assays were not performed with the known substrate L-carnitine and knockdown of hCT2 expression was not analysed for effects on anthracycline resistance. It is noteworthy that the authors examined a few paraffin embedded samples derived from AML patients who were either responsive or unresponsive to anthracyclines in order to determine whether a correlation exists with hCT2 gene expression level, but the results were inconclusive^9^. We subsequently discovered that hCT2 is involved in the uptake of the anticancer drug bleomycin, using cancer cell lines that either expressed or did not express hCT2^11^,^13^,^14^. Moreover, we demonstrated that L-carnitine can effectively block bleomycin uptake and protect cancer cells expressing hCT2 from the genotoxic effects of the drug^11^. In addition to hCT2, OCT1 is another organic cation transporter involved in the uptake of anticancer agents such as platinum drugs^12^,^15^. OCT1 has also been implicated in the transport of the anti-diabetic drug metformin, underscoring the wide range of substrate recognition by these organic cation transporters^16^. Together with the work by Okabe *et al*. (2005) and the above findings, it appears that one or more transporter(s) may be involved in the uptake of anthracyclines into cells.

In this study, we used the high-grade serous ovarian cancer cell line TOV2223G and show for the first time that OCT1 is a high affinity transporter for DNR. Uptake of DNR was effectively blocked by low concentrations of choline, which was previously reported to be a high affinity substrate for OCT1^17^. We also show that shRNA knockdown of OCT1 significantly diminished the expression of the transporter as determined by Western blot analysis probed with anti-OCT1 antibody and greatly reduced DNR uptake in TOV2223G cells. Downregulation of OCT1 enhanced the resistance of these cells to DNR. In support of these observations, expression of OCT1 as an EYFP fusion protein, OCT1-EYFP, revealed that the primary cellular localization of the transporter is on the plasma membrane. These OCT1-EYFP expressing cells increased the uptake of DNR. Collectively, our findings provide compelling evidence that suggests OCT1 functional levels may have a vital role in modulating the response to chemotherapy with anthracyclines.

## Results

### DNR accumulates in several cancer cell lines in a time- and concentration-dependent manner

**Figure 1:**
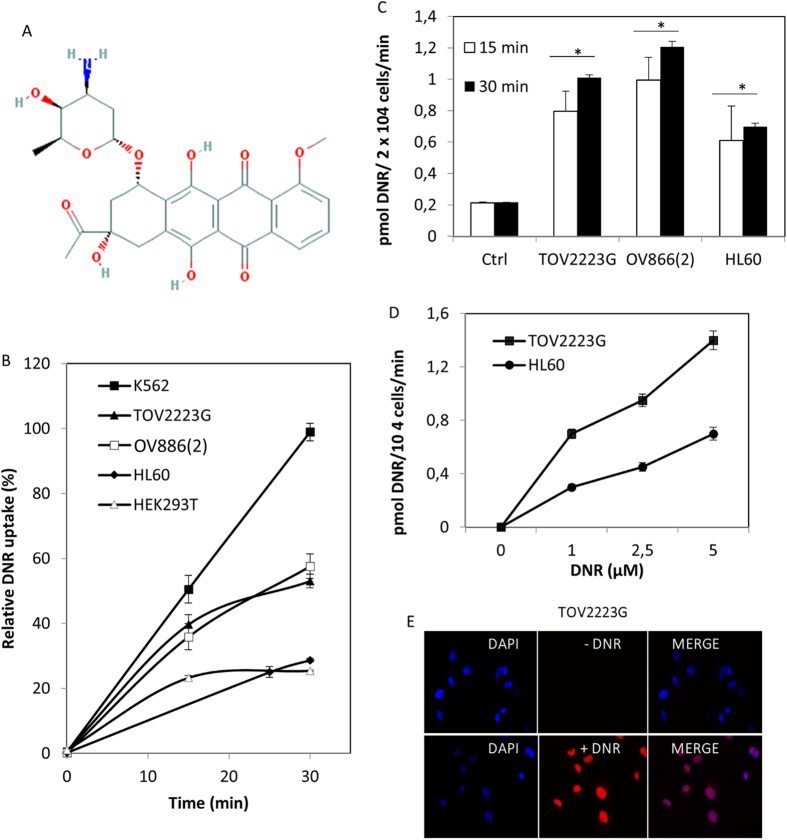
Assessment of DNR uptake in cancer cell lines. After addition of DNR (5 μM) to cells, samples were harvested at the indicated time to monitor for the level of drug uptake. (**A**) Chemical structure of daunorubicin (DNR) obtained from https://www.ncbi.nlm.nih.gov/pccompound?term=daunorubicin. (**B**) Comparison of the relative uptake of DNR into four cancer cells using FACS analysis. Once the drug uptake was stopped, the washed cells were processed for FACS analysis. A total of 10,000 cells were analyzed for DNR fluorescence using FL2 (585/42). Results are expressed as the mean ± S.D. from three separate experiments. (**C**) Fluorescent spectrometry analysis of DNR uptake into TOV2223G, OV866(2), and HL60 cells. The control (Ctrl) are TOV2223G cells without DNR. Increased fluorescence denoting drug uptake was detected with Fluoroskan Ascent Microplate reader set at 544 nm excitation/590 nm emission. The relative fluorescent unit (RFU) representing the measurement of drug uptake was expressed as pmol DNR/2 × 10^4^ cells/min (see Material and Methods). Results are expressed as the mean ± S.D. from three separate experiments. (**D**) Comparison of the concentration-dependent uptake of DNR by TOV2223G and HL60 cells. Cells were incubated with indicated concentrations of DNR then analyzed by the Fluoroskan Ascent Microplate reader. Results are expressed as the mean ± S.D. from three separate experiments. (**E**) Epifluorescent microscopy showing intracellular colocalization of DAPI and DNR in the TOV2223G cell line. Comparisons were made between TOV2223G cells treated with or without DNR, followed by staining of nuclear DNA. Following DNR uptake (5 μM), the cells were washed, fixed with paraformaldehyde and processed for microscopy using Vectashield mounting medium containing 1.5 μg/ml DAPI to detect the nuclear DNA. Images were captured with an Olympus BX51 epifluorescent microscope using Texas Red and DAPI-UV filters at 100× magnification, and then processed with ImageJ software. Merged images overlapped DAPI-stained nucleus (blue) with DNR staining (red). Results are representative of two separate experiments.

Once suitable assay conditions were established (see Materials and Methods), DNR (see chemical structure in Fig. 1A) uptake into cancer cell lines was monitored by three independent methods using either (i) FACS, (ii) a fluorometric microplate reader equipped with a tandem filter set with excitation at 544 nm and emission at 590 nm, or (iii) an epifluorescent microscope. We analyzed DNR uptake in five cell lines that included two of ovarian cancer (TOV2223G and OV866(2)), one of embryonic kidney (HEK293T), and two of leukemia (K562 and HL60). We monitored DNR uptake when the concentration was fixed at 5 μM, a concentration that was used in a study involving ceramide potentiating multidrug resistance^18^ and, in particular, since it was near the estimated concentration of 4 μM given to cancer patients^19^. Each of these cancer cell lines showed a different rate of uptake of DNR with K562 showing the highest level when the concentration was fixed at 5 μM as demonstrated by FACS or fluorometric analysis (Fig. 1B,C, respectively). In addition, DNR entered TOV2223G and HL60 cells in a concentration-dependent manner, with nearly 2-fold more drug accumulating in the TOV2223G cells (Fig. 1D). These data indicate that DNR uptake cannot be by diffusion, otherwise each cell line examined would show the same level of drug uptake (see discussion). Instead, our data suggest that there is an active process to mediate the entry of the drug into these cell lines, which could vary between cell lines.

In parallel, these cells were examined by epifluorescent microscopy which revealed that DNR entered and accumulated in the nucleus (Fig. 1E, and Supplemental data Fig. S1), consistent with the action of the drug in damaging DNA^1^. Accumulation of the drug was not influenced by the cell size as the drug does not stain the cytoplasm, and in addition TOV2223G is significantly larger that K562, which showed the highest level of DNR accumulation. All three methods showed similar results for DNR uptake in a given cell type, although quantitative assessment was achieved by FACS or the fluoro spectrometer.

### A high-affinity transporter mediates DNR uptake in TOV2223G cells

**Figure 2:**
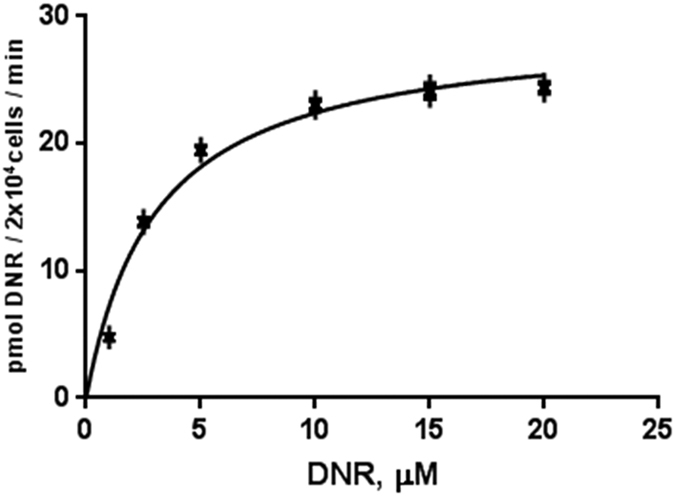
Kinetic analysis reveals a high affinity uptake for DNR into the TOV2223G cells. Uptake rates were recorded for 2.5 min and quantified with the Fluoroskan Ascent microplate reader. The data were fitted to the following equation y = Vmax * x/(Km + x) by means of nonlinear regression using GraphPad Prism (GraphPad Software Inc, CA, USA) to determine Michaelis-Menten constant and maximum velocity. Results are representative of two separate experiments with standard errors.

We selected TOV2223G cells as the model cancer cell line (Fig. 1B) to determine whether DNR transport would be mediated by a low or high affinity transporter. We chose to study the TOV2223G cell line as it showed an intermediate uptake and responded to DNR toxicity (see below), as well as the fact that ovarian cancer patients are also treated with anthracyclines. Kinetic analysis showed that DNR uptake was saturable at ~15 μM (*V*_*max*_ of 29 ± 0.41 pmol/2 × 10^4^/min) with an apparent *K*_*m*_ of 3.0 ± 0.4 μM (Fig. 2). Comparable to the kinetic values reported for L-carnitine transport by the high affinity L-carnitine transporter hCT2^20^, these results indicate that there exists at least one high affinity component for DNR transport into TOV2223G cells, again countering previous arguments that DNR diffuses into cells. Similar kinetics were observed with HEK293T or HL60 cells, yielding apparent *K*_*m*_ of ~5 μM, suggesting that the high affinity DNR transporter also exits in these cancer cell lines. As such, we conducted all subsequent experiments with low concentrations (5 μM) of DNR. In a similar manner, we measured the kinetics for the uptake of another anthracycline, DOX. DOX uptake was also saturable with an apparent *K*_*m*_ of 10 ± 3 μM, suggesting that DNR is a better substrate than DOX for the transporter. In addition, DNR or DOX uptake was not influenced by changes in the pH ranging between 6.5–8.0.

### The organic cation transporters hCT2, OCTN1 and OCTN2 are not involved in DNR uptake

**Figure 3:**
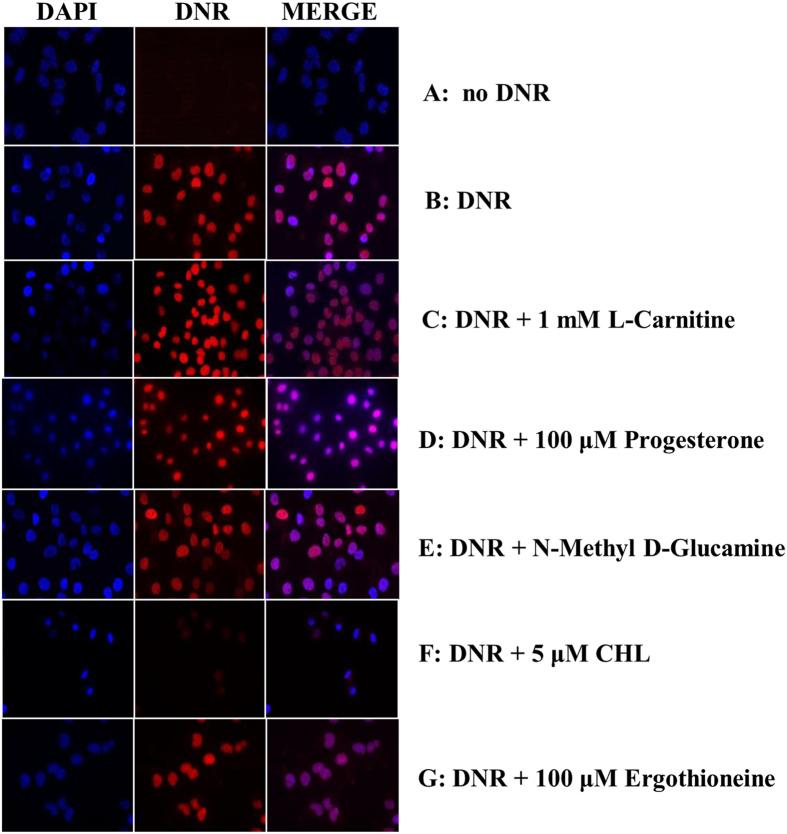
Choline, but not L-carnitine or ergothioneine, serves as a potent competitor in DNR uptake into the TOV2223G cells. The cells were first seeded in 6-well plate onto coverslips at 5 × 10^5^ cells in growth media and allowed to grow overnight. The next day the media was removed and the attached cells were pre-incubated with uptake buffer for 20 min at 37 °C. DNR (5 μM) and selected competitors at the indicated concentrations were mixed together in uptake buffer and added to cells on coverslips, followed by incubation for 1 h. Cells were fixed and images were captured and processed as in Fig. 1E. For panel E, the sodium chloride in the uptake buffer was substituted with N-methyl-D-glucamine. Choline denoted as CHL. Results are representative of at least three separate experiments.

hCT2 and OCTN2 have been discovered as high affinity transporters for L-carnitine, while OCTN1 is reported to be a low affinity transporter for this substrate^10^,^20^,^21^,^22^,^23^. Subsequent studies by Okabe *et al*. raised the possibility that hCT2 and OCTN1 could be transporters for DOX, although no direct experiments were performed to test these assertions^9^,^24^. To determine whether any of these three organic cation transporters are involved in DNR uptake, we examined whether the known substrates for these transporters could compete for the drug uptake. Briefly, a fixed concentration of DNR (5 μM) was added together with a 200-fold excess of L-carnitine (1 mM) to cultures of the TOV2223G and OV866(2) cells preincubated in uptake buffer. As shown in Fig. 3, L-carnitine did not block the uptake of DNR into either of the ovarian cancer cell lines (see also Supplemental data Fig. S2). If indeed hCT2 and OCTN1 were involved in DNR uptake, as previously implicated, we would expect that the excess of L-carnitine to at least cause a significant reduction in DNR uptake. These results indicate that the presence of excess L-carnitine does not affect DNR uptake, suggesting that the three transporters OCTN1, OCTN2 and hCT2 do not mediate DNR uptake. Progesterone, which has been shown to induce hCT2 expression by several fold^25^, did not cause an influx of DNR when cells were pre-cultured in the presence of the hormone for 3 h (Fig. 3D). This is consistent with the above observation that hCT2 does not have a major role in the uptake of DNR.

Since many transporters are known to be Na^+^-dependent or -independent, we examined if omission of NaCl from the uptake buffer would alter DNR uptake in cells. Replacing NaCl with *N*-methyl-*D*-glucamine that maintains the ionic strength in the uptake buffer did not affect DNR entry into cells (Fig. 3E), suggesting that transport of DNR into the cells is not coupled with the movement of Na^+^ ions.

### Choline effectively blocks DNR uptake

**Figure 4:**
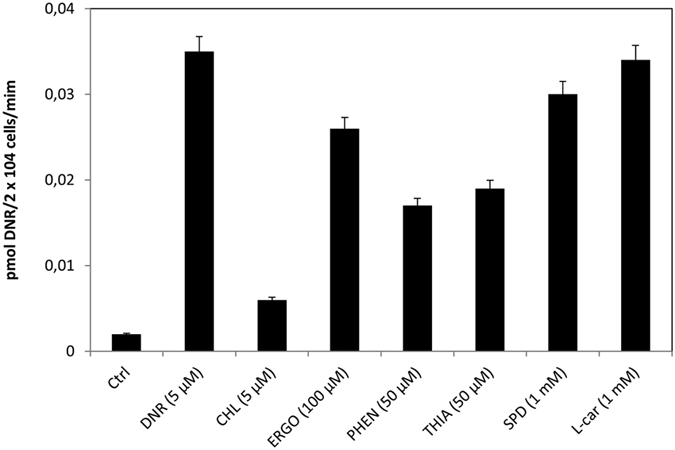
Phenformin and thiamine partially inhibit DNR uptake in TOV2223G cells. Cells were seeded in 96-well plates and DNR uptake was monitored as in Fig. 1C, but when added together with either choline (CHL), ergothioneine (ERGO), phenoformin (PHEN), thiamine (THIA), spermidine (SPD) or L-carnitine (L-car). Cells without DNR were designated as the control (Ctrl). Results are expressed as the mean ± S.D. from three separate experiments.

We next examined whether the OCT family members of polyspecific organic cation transporters would be involved in mediating DNR uptake. OCT1, 2 and 3 are reported to have different affinities for a wide range of cationic substrates that include choline, ergothioneine, phenformin (a structurally related form of metformin), thiamine and polyamines^17^,^26^,^27^. Incubation of the TOV2223G cells with DNR together with the indicated cationic substrate, revealed that choline effectively competed with DNR for uptake, whereas ergothioneine (100 μM), phenformin (50 μM), and thiamine (50 μM), partially (p < 0.01) inhibited DNR uptake at the concentrations used (Figs 3F,G and 4, respectively). In contrast, a high concentration (1 mM) of polyamine spermidine (SPD) had only a modest effect on the uptake of DNR (Fig. 4). This is consistent with OCT transporters that function as low affinity permeases for polyamines^28^. Since choline is a potent competitor in DNR uptake and OCT1 has been previously described as a high affinity transporter of choline, our results suggest that OCT1 may play a role in the high affinity uptake of DNR.

### shRNA-OCT1 knockdown in TOV2223G cells reduces DNR uptake and enhances resistance to the drug

**Figure 5:**
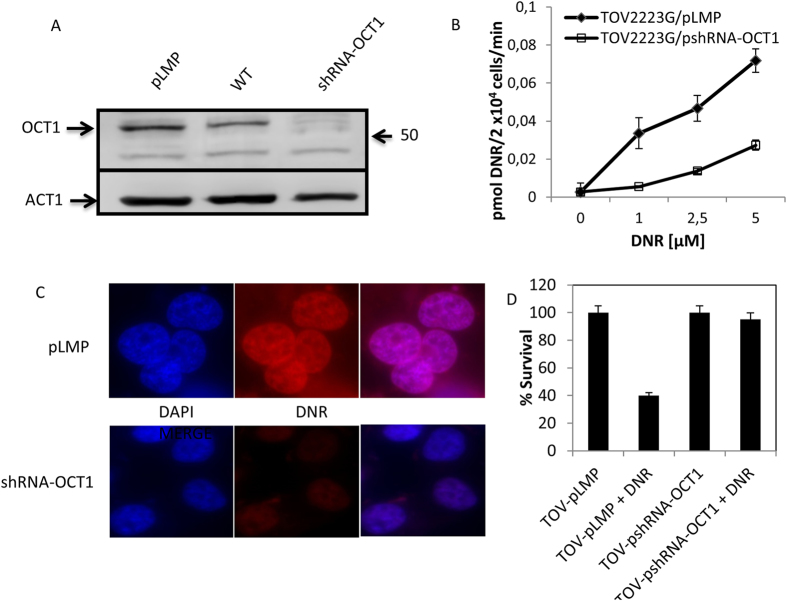
shRNA-OCT1 downregulation in TOV2223G cells diminishes DNR uptake and confers resistance to the drug. (**A**) Western blot analysis. Total cell extracts were prepared from stable clones of TOV2223G cells carrying either the empty vector pLMP or the plasmid pshRNA-OCT1 and processed for Western Blot analysis using anti-hOCT1 antibody. Each lane contained 30 μg of total protein extract. Actin (ACT1) was used as the internal control. WT is the TOV2223G cells alone. Results are representative of three separate experiments. (**B**) Comparison of the concentration-dependent uptake of DNR into stable clones carrying either pLMP or pshRNA-OCT1. DNR uptake was monitored using the Fluoroskan Ascent microplate reader as above. Results are expressed as the mean ± S.D. from three separate experiments. (**C**) Epifluorescent microscopy reveals that OCT1 downregulation severely inhibits DNR uptake into TOV2223G cells. The stable clones of TOV2223G carrying either pLMP or the knockdown plasmid pshRNA-OCT1 were fixed with PFA, and processed for microscopy, using Axio Imager Z2 with Texas Red and DAPI-UV filters at 63× magnification. Images were processed with AxioVision software. Results are representative of three separate experiments. (**D**) Cell survival upon exposure to DNR. The TOV2223G cells and stable clones carrying either the empty vector pLMP or pshRNA-OCT1 were exposed to DNR (5 μM) for 72 h, and the viability of the cells was monitored using MTT assay. Results are expressed as the mean ± S.D. from two separate experiments.

It has been shown that OCT1 is expressed predominantly in the liver, the organ that metabolizes DNR, whereas expression levels of OCT2 and OCT3 are not detected^29^. To test the hypothesis that OCT1 is a DNR transporter, we designed two shRNA constructs to specifically downregulate the transporter in the TOV2223G cells. These constructs targeted the 5′-untranslated and the C-terminus of the mRNA sequence of the OCT1 gene. Stable clones carrying the shRNA construct against the 5′-untranslated region was effective in down-regulating OCT1 expression levels by more than 95% when compared to the control shRNA LMP vector, as determined by immunoblot analysis probed with polyclonal antibody against OCT1 and quantified by Image J (Fig. 5A). The shRNA construct against the C-terminus of OCT1 had no effect in downregulating OCT1 expression (data not shown). Consistent with the downregulation of OCT1, fluorometric analysis revealed that DNR uptake was significantly reduced (3- to 5-fold) in the shRNA-OCT1 knockdown cells compared to cells carrying the control shRNA (Fig. 5B). The low level of DNR uptake observed with the shRNA-OCT1 knockdown cells demonstrates that downregulation by shRNA-OCT1 was not sufficient to abolish DNR transport or that gene knockdown was incomplete, although other redundant transporter may exist to mediate DNR uptake. In a parallel experiment, epifluorescent microscopy showed that the accumulation of DNR was nearly undetectable in the shRNA-OCT1 knockdown cells compared to the shRNA LMP vector control cells (Fig. 5C). There was no difference in DNR uptake between the shRNA LMP vector control and the TOV2223G cells alone (data not shown), suggesting that the slightly higher OCT1 protein level detected in the LMP vector control vs the TOV2223G cells could be due to susceptibility to extraction (Fig. 5A). In another control experiment, the uptake of rhodamine was not altered in the shRNA-OCT1 knockdown cells compared to the shRNA control cells (see also below). These results strongly support a role for OCT1 in mediating DNR uptake into TOV2223G cells.

To investigate the effect of OCT1 knockdown with respect to cell sensitivity to DNR, we performed a viability assay to monitor surviving cells, following exposure to the drug for 72 hours. Under these conditions, the normal TOV2223G cells carrying the LMP vector showed a survival of nearly 40% (Fig. 5D). However, OCT1 knockdown TOV2223G cells were protected from DNR toxicity and showing nearly 100% survival (Fig. 5D). This finding strongly supports a role for OCT1 in the transport of DNR, and excluding any possibility of drug diffusion into the cells.

### OCT1-EYFP overexpression enhances DNR uptake in HEK293T cells

**Figure 6:**
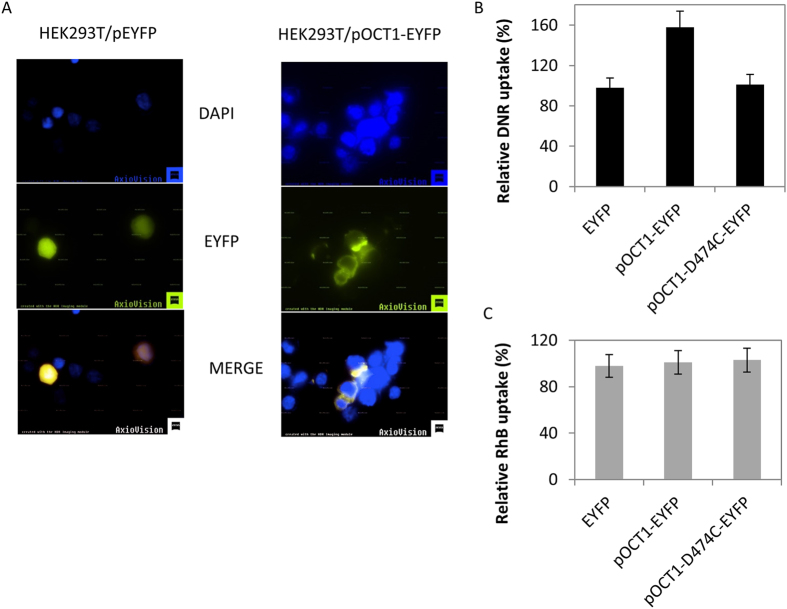
Expression of OCT1-EYFP, but not the OCT1-D474C variant, increases DNR uptake in HEK293T cells. The plasmid pOCT1-EYFP was used to create the OCT1-D474C mutation by site-directed mutagenesis. The plasmids pEYFP (empty), pOCT1-EYFP and pOCT1-D474C-EYFP were transiently transfected into HEK293T cells and exposed to DNR. Cells expressing EYFP were sorted to quantify the level of DNR uptake using FACS analysis using FL1 530/30 to detect EYFP fluorescence and FL4 670LP for DNR fluorescence. (**A**) Epifluorescent microscopy showing localization of the OCT1-EYFP fusion protein. Pictures were taken with Ziess Z2 fluorescent microcope using DAPI filter set and EYFP filter set. Results are representative of three separate experiments. (**B**) DNR uptake in HEK293T cells carrying either the plasmid pOCT1-EYFP, the variant, or the empty EYFP vector. Results are expressed as the mean ± S.D. from three separate experiments. (**C**) Comparison of the rhodamine uptake level in HEK293T cells carrying the empty EYFP vector, or either the plasmid pOCT1-EYFP or pOCT1-D474C-EYFP. Results are expressed as the mean ± S.D. from three separate experiments.

To determine whether OCT1 overexpression could enhance DNR uptake in cells, we designed an expression system to drive OCT1 expression as a OCT1-EYFP fusion protein in the vector pEYFP-N1^30^. Transient transfection with pOCT1-EYFP into the TOV2223G cell line yielded very few cells that expressed the OCT1-EYFP fusion protein (<1% in the transfected population). Therefore, we used HEK293T cells as an alternative host cell since it was reported to accommodate the expression of plasma membrane transporters^27^,^31^. At least 5–10% of the pOCT1-EYFP transfected HEK293T cells expressed the OCT-1 EYFP fusion protein. Epifluorescent microscopy revealed that the OCT1-EYFP protein was specifically present on the plasma membrane, consistent with a previous report that used immunopurified antibodies against OCT1^27^,^31^, while the EYFP alone showed non-specific cellular localization (Fig. 6A). Because HEK293T cells can accommodate the ectopic expression of the OCT1-EYFP fusion protein, we monitored the uptake level of DNR into these cells. After 24 hours of transient transfection of HEK293T cells with the empty pEYFP-N1 vector or the pOCT1-EYFP plasmid, the cells were preincubated in uptake buffer followed by the addition of DNR (5 μM) for 1 hour and uptake was determined by FACS analysis only in the fraction of EYFP expressing cells (see Materials and Methods). The OCT1-EYFP expressing cells showed nearly 50% increase in DNR uptake, compared to the cells that were transfected with the empty vector alone (Fig. 6B). In contrast, OCT1-EYFP expression did not stimulate rhodamine uptake in HEK293T cells (Fig. 6C). These findings show that the expression of OCT1-EYFP fusion protein correlates with enhanced DNR uptake, and strongly reinforces the role of OCT1 as a transporter of DNR.

### The variant OCT1-D474C-EYFP is unable to increase DNR uptake in HEK293T cell**s**

Molecular models have predicted that the transmembrane helix domain 11 of the rat OCT1 is involved in substrate binding^32^. Several mutations have been created in this region of the rat OCT1 gene^32^. In particular, the replacement of Asp475 with cysteine resulted in nearly complete reduction (98%) of the transport of the model substrate tetraethylammonium in oocytes expressing the rOCT1-Asp475Cys variant^32^. The same mutation was created in the OCT1-EYFP construct by site-directed mutagenesis; Asp474, which corresponded to the amino acid residue of the human OCT1, was substituted with cysteine generating the variant OCT1-D474C-EYFP. Transient expression of this variant in HEK293T cells demonstrated its localization to the plasma membrane (Supplemental data Fig. S4). However, this OCT1-D474C variant did not enhance DNR uptake in HEK293T cells, as observed with the native OCT1-EYFP construct (Fig. 6B). We conclude that the transport function of OCT1 is required for the uptake of DNR and exclude the possibility that OCT1 might act *via* another transporter through protein-protein interaction.

### High levels of OCT1 mRNA correlates with increased survival in patients with high-grade serous epithelial ovarian cancer

**Figure 7:**
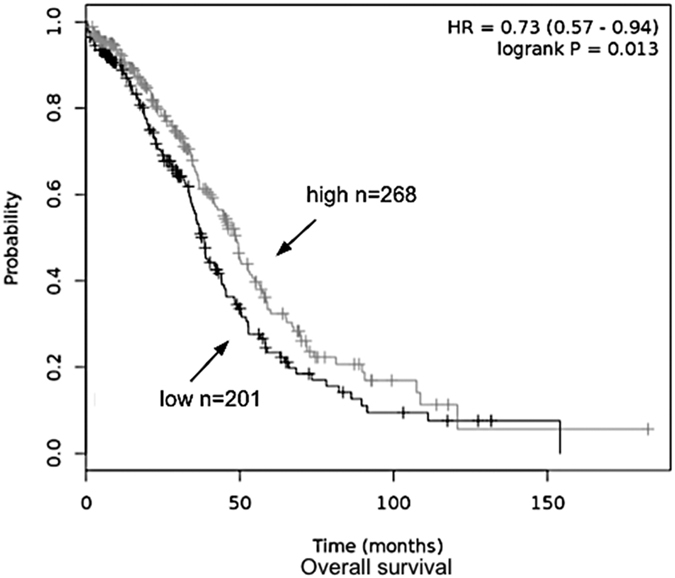
High OCT1 mRNA levels are associated with better overall survival of high-grade serous epithelial ovarian cancer patients. Overall survival plot was generated online using the Kaplan-Meier Plotter based on signal intensity of the SLC22A1 probe (207201_s_at) in Affymetrix microarray gene expression data from high-grade serous epithelial ovarian cancer patients of The Cancer Genome Atlas. Auto select best cutoff was chosen in the analysis; cutoff value was 19 and expression range of the probe was 1–150. A total of 469 patients were available and samples were split in two groups (high and low) according to the cutoff value. The hazard ratio with 95% confidence intervals and log rank *p* value was calculated and significance was set at *p* < 0.05.

To explore the potential significance of OCT1 in the context of chemotherapy, we used the Affymetrix gene expression to analyze 469 cases of high-grade serous epithelial ovarian cancer from The Cancer Genome Atlas (TCGA) dataset^33^. The dataset represented patients in different stages of the cancer and were treated with a combination chemotherapy that included platinum and taxol reagents. Using the online tool Kaplan-Meier Plotter^34^,^35^, we observed that patients with a high level of OCT1 mRNA have significantly better overall survival (*p* = 0.013) than those with low expression levels of this gene (Fig. 7). This difference accounts for an average of 10 months increased life expectancy for patients with higher OCT1 expression levels (Fig. 7). Since OCT1 has the ability to transport derivatives of cisplatin, as well as paclitaxel^12^,^36^, it is possible that ovarian cancer patients expressing reduced levels of OCT1 are associated with poor outcome following chemotherapy.

## Discussion

In this study, we demonstrate that the human organic cation transporter OCT1 is involved in the high affinity transport of DNR, a member of the anthracycline family of anticancer drugs. This conclusion was derived from the following findings: (i) several cell lines displayed saturable transport of DNR; (ii) the kinetics for DNR transport is comparable for the uptake of the model substrate tetraethylammonium and the neurotoxin methyl-4-phenylpyridinium with apparent *K*_*m*_ ranging between 5–50 μM by HEK293 cells designed to express the human OCT1^32^,^37^; (iii) the rate of DNR uptake varies between cell lines and may be dependent upon the expression level of the OCT1 transporter (see Supplemental data Fig. S3); (iv) choline at low concentrations effectively competed with DNR uptake, underscoring the participation of a high affinity choline uptake system in the transport of DNR; (v) downregulation of OCT1 reduced the transport of DNR into the cancer cell line TOV2223G, as well as causing DNR resistance; and (vi) expression of OCT1 as an EYFP fusion protein revealed that it is localized to the plasma membrane consistent with a previous report^27^,^31^, and enhanced the uptake of DNR, but not rhodamine. In this latter finding, not all of the expressed OCT1-EYFP protein might be functionally active to yield substantially higher level of DNR uptake. The overexpression of OCT1-EYFP might displace resident plasma membrane protein that could have adverse effects on the functioning of the transporter such as causing the eviction of accessory proteins that are necessary for the proper transport function of OCT1^38^. On the basis of our findings, we exclude the possibility that DNR uptake can be explained by the drug diffusing into the cells. In fact, there is growing evidence precluding a diffusion process by which DNR enters into cells. An earlier study demonstrated that DOX can penetrate artificial membranes, but it cannot readily go through natural membranes^39^. Moreover, yeast cells lacking the plasma membrane regular Agp2 that controls the expression of the uptake transporters Sam3 and Dur3 do not take up DOX^40^. Uptake of DOX into the *agp2*Δ mutant can be rescued by the expression of either of the active transporters Sam3 or Dur3, which provides strong support for transporter-mediated uptake of anthracyclines into cells as the principal process^40^.

In the OCT family of transporters, OCT1 shares 75 and 50% identity at the amino acid level with OCT2 and OCT3, respectively. We propose that OCT1 has a major role as a transporter for anthracyclines. The deletion of the OCT1 gene in mice caused significant upregulation of OCT2 and OCT3^17^,^26^,^27^, suggesting that if a similar mechanism occurs with OCT1-shRNA downregulation in human cells then these two later transporters are unlikely to be involved in DNR uptake since they cannot compensate for OCT1 role in the drug uptake. Notwithstanding, we have not directly tested whether OCT2 and OCT3 could perform a role in DNR transport. While these three transporters share the ability to transport metformin and the model substrate tetraethylammonium, they also mediate the uptake of distinct substrates^27^,^31^. OCT2 has been shown to transport platinum anticancer drugs and both OCT2 and OCT3 secrete small organic cations in the kidney and placenta. OCT2 and OCT3 also play important roles in the brain by transporting neurostimulants such as dopamine, corticosterone, amphetamine and methamphetamine^29^,^41^. Thus, these two transporters have an important role in the neurological response to psychostimulants, and their expression is strongly linked to addiction behavior^29^,^42^. Since anthracyclines undergo metabolism in the liver where OCT1 is expressed, as well as OCT3, but not OCT2^43^, raises the possibility that at least OCT2 may not be involved in the uptake of anthracyclines and that substrate specificity may be governed by the site of action. Thus, the residual level of DNR uptake in the TOV2223G cells knockdown for OCT1 could be accounted for if there is at least one other DNR transporter.

OCT1 also shares nearly 30% identity at the amino acid level with transporters that belong to the OCTN family, which include OCTN1, OCTN2 and OCT6/CT2^29^. OCTN1 is a low affinity transporter for L-carnitine, but a high affinity transporter for ergothioneine, while both OCTN2 and CT2 are high affinity transporters for L-carnitine^20^,^21^,^23^. L-carnitine in excess of 200-fold did not block DNR uptake, thereby excluding these three transporters in the uptake of anthracyclines. This is in stark contrast to the previous report by Okabe *et al*. which described CT2 as a high affinity transporter for DOX, based on limited data from *X. laevis* oocytes injected with hCT2 cRNA, and *K*_*m*_ measurements for DOX uptake^9^. It remains inconclusive whether hCT2 cRNA produced a modified protein that indirectly promoted high affinity uptake of DOX since inhibition of DOX uptake and CT2 knockdowns were not conducted in their study^9^. However, there is additional evidence that excludes the OCTN family as transporters of anthracyclines. The leukemia cell line K562 does not express CT2 (Supplemental Fig. S5), yet these cells allow entry of DNR, thus eliminating the possibility that CT2 is involved in the transport of this drug^44^. Moreover, the HL60 cells expressed the CT2 transporter, but exhibited the slowest rate of DNR uptake. It is noteworthy that the HL60 cells weakly expressed the OCT1 protein, while it is highly expressed in the K562 cells (Supplemental Fig. S3). This observation is consistent with the notion that DNR uptake is directly proportional to the expression level of the OCT1 transporter.

We note that OCTN1 was described previously as a high affinity transporter of ergothioneine^23^ and subsequently, as a potential transporter of DOX based on gene expression analysis data from the NCI-60 panel of cell lines^24^. However, when the TOV2223G cells were challenged with a 200-fold excess of ergothioneine, there was only a partial effect on the uptake of DNR. Therefore, OCTN1 does not appear to have a major role in anthracycline uptake. Our observation of partial inhibition of DNR uptake by ergothioneine may be explained if human OCT1 also has a role in the uptake of this antioxidant. This is consistent with reports describing *C. elegans* OCT-1 as a transporter for ergothioneine, and more recently, as a transporter for DOX^26^,^40^.

Of the various competitors tested in this study, choline, an important component for the biogenesis of the plasma membrane and synthesis of the neurotransmitter acetylcholine^45^, was the most effective in abolishing DNR uptake. This is consistent with a previous report demonstrating that OCT1 displayed the ability to transport choline^17^. This raises the issue whether high affinity choline transporters in general have the ability to transport DNR. Three different transport systems participate in choline uptake: the high affinity choline transporter 1, CHT1; the choline transporter like 1, CTL1; and the OCTs such as OCT1 and OCT2^45^. CHT1 has the highest affinity (*K*_*m*_ of 1 μM) for choline uptake, while CTL1 is in the range of 25–50 μM and OCT1 at nearly 42 μM^17^. The CHT1 transporter acts exclusively in neurons where it transports acetylcholine in a sodium-dependent manner. Since the uptake of DNR is independent of sodium, it suggests that CHT1 is unlikely to be involved in DNR uptake. CTL-1 primarily transports choline in non-neuronal cells independent of sodium^46^. As such, both CTL-1 and OCT1 share common properties, however we did not examine whether shRNA against CTL-1 would further diminish the uptake of DNR in TOV2223G shRNA-OCT1 cells that were downregulated for OCT1 expression. Other possibilities might exist to account for our finding that very low concentrations (0.5–5 μM) of choline can strongly compete for DNR uptake, if indeed both CTL-1 and OCT1 operates at low *K*_*m*_. One possibility is that a high affinity sensor could sense the low level of choline and in turn negatively regulates the activity of the lower affinity choline transporters such as OCT1 in a manner that has been reported for transceptors^47^.

Our findings could have direct impact on the chemotherapy regimen that is given to ovarian cancer patients. There are four major types of primary ovarian adenocarcinoma and these are categorized as serous, mucinous, endometrioid, and clear-cell. Serous adenocarcinoma comprises about one half of all ovarian cancers. Currently, the combination of paclitaxel and cisplatin is the standard regimen for ovarian cancers with a response rate of nearly 70%^48^. This combination of paclitaxel-platinum chemotherapy appears to improve overall and disease-free survival not only for patients with primary ovarian cancer, but also for patients with relapsed disease^49^. Although treatment of this disease has improved, the 5-year survival of patients with advanced-stage ovarian cancer is less than 20%, and there is an ongoing effort to improve the outcome of treatment of ovarian cancer. There is evidence that addition of DOX to the paclitaxel-platinum combination regimen can significantly improve survival of ovarian cancer patients^50^,^51^,^52^. However, the actual molecular mechanism leading to improved survival is not known. Here, we propose that the expression level of OCT1 could dictate the added benefits for ovarian cancer patients receiving DOX together with the paclitaxel-platinum regimen, or to substitute paclitaxel with DOX in light of the observation that OCT1 can also transport paclitaxel^36^. This is in accordance with our results showing a better overall survival in high-grade serous ovarian cancer patients having high OCT1 mRNA levels, which could mediate increasing uptake of both platinum and paclitaxel (Fig. 7)^12^,^15^,^36^. OCT1 could also play a pivotal role in the outcome of other diseases that are treated with anthracyclines. For example, patients with AML, a major cause of mortality from hematological malignancies in adults, are given a standard induction chemotherapy consisting of an anthracycline, such as DNR^53^. Importantly, a significant fraction (>50%) of older AML patients (>60 yrs) do not achieve complete remission after the induction chemotherapy with DNR, and in some cases, remission lasts for a short duration due to drug resistance^3^,^4^,^5^. The underlying mechanisms that are responsible for DNR resistance in these AML non-responding patients are not known, although it is possible that one of the mechanisms could be due to a defect in the entry of the drug into the cancer cells. Whether there are mutations that disrupt the function of OCT1 in a fraction of the AML non-responders, remains to be explored. It should be noted that there are genetic polymorphisms in the OCT1 gene that altered, e.g., metformin uptake (https://www.pharmgkb.org/). However, it is cumbersome to recreate the various mutations to test for alteration in DNR uptake, and instead we are screening DNR unresponsive AML patients for defective drug uptake and then establish whether these patients have mutations in the OCT1 gene.

## Materials and Methods

### Cell lines and cell culture

TOV2223G and OV866(2) were obtained from the RRCancer-Biobanque de cancer de l’ovaire du CHUM and are spontaneously immortalized cell lines derived from high-grade serous ovarian cancer patients’ solid tumor or ascites, respectively, and previously described^54^,^55^. Cell lines were grown in OSE growth medium (Wisent, St-Bruno, QC) supplemented with 10% FBS, 2 mM L-glutamine, 0.5 μg/mL amphotericin B and 50 μg/mL gentamycin at 37 °C, 5% CO_2_, 95% Air. The leukemia cell lines HL60 and K562 were purchased from ATCC and kindly provided by Dr. Denis-Claude Roy (Maisonneuve-Rosemont Hospital research center) were grown in RPMI 1640 supplemented with 10% FBS, 2 mM L-glutamine, 100 U/mL penicillin and 100 μg/mL streptomycin at 37 °C with 5% CO_2_. The embryonic human kidney cell line HEK293T was kindly provided by Dr. El Bachir Affar (Maisonneuve-Rosemont Hospital research center) and grown in DMEM medium supplemented with 10% FBS, 2 mM L-glutamine, 100 U/mL penicillin and 100 μg/mL streptomycin at 37 °C with 5% CO_2_.

### Antibodies, cDNA, vectors, and drugs

The SLC22A1 (NBP-59464) and β-Actin (AC-15) antibodies were purchased from Novus and Santa Cruz, CA, USA, respectively. hSLC22A1 cDNA (RDC0425) was purchased from R&D Systems. The MSCV-LTRmiR30-PIG (LMP) vector was purchased from Thermo Scientific and pEYFP-N1 (6006–1) was purchased from Clontech. Daunorubicin was purchased from Maisonneuve-Rosement Hospital, Montreal, Canada.

### Generation of hOCT1 shRNA for OCT1 knock down in TOV2223G cells

hOCT1 knock down was generated by two shRNA sequences obtained from the RNAi Central- RNAi Oligo Retriever (http://katahdin.cshl.org:9331/homepage/siRNA/RNAi.cgi?type=shRNA; hannonlab.cshl.edu/GH_shRNA.html;) and listed in supplemental Table S1. Among the two generated sequences, sequence 1 was able to most efficiently silence hOCT1 protein expression and was chosen for all subsequent studies. The sense and antisense strands were linked with miR30 loop sequence and the miR-30 styled sequence was synthesized as a single stranded DNA oligonucleotide with one part corresponding to the endogenous miR-30 miRNA flanking sequence. The resulting 97-mer was PCR amplified using common miR30 primers listed in supplemental Table S2 and subcloned into the EcoRI/XhoI sites of MSCV-LTRmiR30-PIG (LMP). This generated the hOCT1 shRNA plasmid.

### Generation of hOCT1-EYFP fusion protein and the D474C variant

Human SLC22A1 cDNA plasmid (RDC0425, R&D Systems, USA) containing the hOCT1 cDNA isoform 1 (1678 bps) was used as template for PCR amplification with the primers listed in supplemental Table S2. The PCR product was subcloned into the XhoI/EcoRI sites of the mammalian expression vector, pEYFP-N1. The resulting hOCT1-EYFP construct was amplified and positive clones were sequenced using sequencing primer. The hOCT1-D474C-EYFP variant was created by site-directed mutagenesis using the primers listed in supplemental Table S2.

### DNR uptake assay

The day of the experiment, media was removed; cells were then washed with 2 mL Uptake buffer (125 mM NaCl, 10 mM HEPES pH 7.4, 5.6 mM glucose, 4.8 mM KCl, 1.2 mM KH_2_PO_4_, 1.2 mM CaCl_2_, 1.2 mM MgSO_4_) for 5 min at gentle shaking and incubated in 1.9 mL of the same buffer for 20 min. Drug uptake was initiated by the addition of 5 μM DNR. DNR (5 mg/mL equivalent to 8.86 mM) was kept as a stock solution in water at −20 °C and diluted to 100 μM in uptake buffer. Uptake was monitored over time, most often for 60 min at 37 °C. For competition studies, the competitor was mixed together with 5 μM DNR and then added to the cells in the uptake buffer. Uptake was stopped by directly adding 2 mL ice cold uptake buffer to the drug-buffer complex or the drug-competitor-buffer complex which was immediately removed, followed by three successive washes with 2 mL of ice cold uptake buffer with gentle shaking for 5 min each. Cells were then collected by adding 0.25% Trypsin-EDTA to detach adherent cells (TOV2223G, OV866(2) and HEK293T) and centrifuged at 1000 × g for 5 min.

### Fluoroskan analysis

Cells treated as described were resuspended in 1 mL Uptake buffer prior to cell count or directly spin in the case of suspension cells HL60. Cells (2 × 10^5^) were resuspended in 1 mL of Uptake buffer and 100 μL of resuspension (2 × 10^4^) was added to each well in triplicate in a 96-well black well with optical bottom (Fisher Scientific). DNR cellular uptake was monitored with a microplate fluorometer (Fluoroskan Ascent, Thermo Scientific, USA) using tandem filter 544 nm excitation and 590 nm emission.

### Flow cytometry analysis

Cells were fixed with 100 μL of 4% paraformaldehyde (PFA) for 10 min. Total cells were centrifuged to be resuspended in PBS (300–500 μl depending on the pellet size) then DNR cellular uptake was measured by FACScalibur, Becton-Dickinson (San Jose, CA). In the case of simple DNR uptake assessment, 10 000 cells were analyzed with FL2 (585/42) as described previously^40^. In the case of overexpression with EYFP tag, 55 000 cells (or total sample) were analyzed and cells expressing EYFP fluorescence were gated and the DNR fluorescence level was measured only for these EYFP expressing cells. For EYFP FL1 (530/30) was used and FL4 (670LP) was used for DNR fluorescence detection.

### Epifluorescence analysis for DNR uptake

Cells were grown in 6-well plates containing 18 × 18 − 1 coverslips in 2 mL OSE complete medium and left to recover for 16–24 h. The cells were treated with DNR as previously described then fixed with 100 μL of 4% PFA for 10 min. After fixation, cells were washed three times as described previously and mounted with 5 μL of mounting media (Vectashield, Vector Laboratories Inc., CA, USA) containing 1.5 μg/mL of 4′,6-diamidino-2-phenylindole (DAPI) on a microscope slide, and sealed with nail polish. Images were photographed with the Zeiss Z2 imager or the Olympus BX53 fluorescent microscope at 63× and 60× magnification, respectively, using DAPI/Texas Red filters. Images were then processed with Axio Vision (Zeiss Z2) or ImageJ (Olympus BX53) software.

### Epifluorescence analysis for hOCT1-EYFP overexpression

After overnight transfection in 6 well plate, the media was removed and HEK293T were briefly washed in PBS then collected by adding 0.25% Trypsin-EDTA. Cells were centrifuged and wash once in 1 ml PBS then fixed with 100 μL of 4% PFA for 10 min. After fixation, cells were washed once in PBS and resuspended in 30 μL of Vectashield mounting media containing 1.5 μg/mL 4′,6-diamidino-2-phenylindole (DAPI) (Vector Laboratories Inc., CA, USA). 7,5 μl of the suspension were put on a microscope slide. Images were photographed with Zeiss Z2 imager at 63× magnification using DAPI/EYFP filters. Images were then processed with Axio Vision software.

### shRNA transfection

TOV2223G cells were grown in 6-well plate in OSE complete media containing 10% FBS and antibiotics. When cells reached 60–70% confluence, media was replaced with fresh OSE media containing 10% FBS, but no antibiotics. 2.5 μg hOCT1 shRNA pDNA were mixed with 7.5 μL Lipofectamine 2000 (Life Technologies) in ratio 1:3. LMP shRNA pDNA was used as control vector. Cells were selected with Puromycin (2 μg/mL) for stable clones for 10 days before analysis.

### Transfection of HEK293T cells for epifluorescence analysis

HEK293T cells at 60–70% confluence on coverslips in a 6-well plate were transfected overnight with one of three plasmids EYFP-N1, hOCT1-EYFP, hOCT1-D474C-EYFP and Lipofectamine 3000, as per manufacturer’s instructions. Next day, they were processed for either DNR uptake assay analyzed by flow cytometry or fluorescence microscopy analysis.

### Rhodamine B (RhB) uptake assay

Transfected HEK293T cells were processed the same way as for DNR uptake assay but the 5 μM DNR were replaced by 50 μM RhB and FL2 (585/42) was used to detect RhB fluorescence in FACS analysis of the samples

### Western Blot Analysis

Equal amounts of whole cell extract protein (30 μg) were separated by sodium dodecyl-polyacrylamide (SDS) polyacrylamide gel electrophoresis. Proteins were then transferred onto PVDF membrane and immunoblotted with anti-hOCT1 antibody at a 1:1000 dilution in PBST buffer (137 mM NaCl, 2,7 mM KCl, 10 mM Na2HPO4, 1,8 mM KH_2_PO_4_, 0,1% Tween 20). Equal protein loading was confirmed by reprobing the membrane with anti-β-Actin.

### MTT Assay

TOV2223G pLMP control vector and TOV2223G hOCT1 shRNA were seeded in a 96-well plate. Each well contained 7 × 10^3^ cells per 100 μl with 5 μM DNR. After 72 hours of incubation, 20 μL of Methylthiazolyldiphenyl-tetrazolium bromide (MTT; Sigma-Aldrich, Ltd, Ontario, Canada) concentrated at 5 mg/mL in uptake buffer, was added to each well and incubated for another 3 h at 37 °C. The reaction was stopped by adding DMSO (Sigma lifesciences, Canada) in each well to dissolve the MTT crystals. Conversion of tetrazole into purple formazan product was detected at 540 nm, using the ELx808 Absorbance Microplate Reader (Biotek, Winooski, USA). All experiments were performed in triplicate.

### Kinetic studies

TOV2223G cells were seeded at 2 × 10^4^ cells per 100 μL with increasing concentrations of DNR. Initial rates were obtained by measuring uptake at 2.5 min, using the Fluoroskan Ascent Microplate reader set at 544 nm excitation/590 nm emission tandem filter. Measurements were expressed in relative fluorescence units (RFU). To estimate the kinetic parameters, uptake rates were fitted to the equation y = Vmax * x/(Km + x), by means of nonlinear regression using GraphPad Prism(GraphPad Software Inc, CA, USA) in which Km and Vmax are Michaelis-Menten constant and maximum velocity respectively. The recorded RFUs were first multiplied by 1 × 10^−4^ L (100 μL), the total volume of buffer used to stop uptake, and then divided by 2.5 (time for uptake), and finally multiplied by 10^6^ to give pmole DNR/2 × 10^4^ cells/min.

### Kaplan-Meier plotter analysis

The prognostic value of the SLC22A1 gene (hOCT1 protein) in high-grade serous epithelial ovarian cancer was analyzed using the online tool Kaplan-Meier Plotter (http:/kmplot.com/analysis/), a database that integrates gene expression data and clinical information of breast, ovarian, lung and gastric cancers^34^,^35^. Kaplan-Meier Plotter uses three versions of the Affymetrix HG-U133 datasets (with 22,277 probe sets in common), and clinical data from Gene Expression Omnibus (GEO) and The Cancer Genome Atlas (TCGA) datasets. The expression of SLC22A1 in the TCGA dataset for high-grade serous epithelial ovarian cancer was verified with the best specific probe (JetSet probes) of this gene (207201_s_at). A total of 469 patients were available for analysis on overall survival. Patient samples were split into two groups according to the median value, using the query parameter of auto-select best cutoff. The signal range of the SLC22A1 probe was 1–150, and the cutoff value was 19. The two patient groups (high and low expression levels) were compared using a Kaplan-Meier survival plot. The hazard ratio with 95% confidence intervals and log rank *p* value was calculated, and significance was set at *p* < 0.05.

## Additional Information

**How to cite this article**: Andreev, E. *et al*. The human organic cation transporter OCT1 mediates high affinity uptake of the anticancer drug daunorubicin. *Sci. Rep.* **6**, 20508; doi: 10.1038/srep20508 (2016).

## Supplementary Material

Supplementary Information
