# Supplementary material for: The human organic cation transporter OCT1 mediates high affinity uptake of the anticancer drug daunorubicin
Source: Sci Rep. 2016 Feb 10;6:20508. doi: 10.1038/srep20508 (PMC4748219; doi:10.1038/srep20508)
Supplement: Supplementary Information [file srep20508-s1.doc]

**The human organic cation transporter OCT1 mediates high affinity uptake of the anticancer drug daunorubicin**

Emil Andreev, Nicolas Brosseau, Euridice Carmona, Anne-Marie Mes-Masson and Dindial Ramotar

**Supplemental Table 1.** The short hairpin sequences used in this study

| **Sequence no** | | **sh sense sequence 5’-3’** | **sh entire sequence**  **5’-3’** |
| --- | --- | --- | --- |
| 1 | UGGCCACUGCCUGAUUUCAAAC | | TGCTGTTGACAGTGAGCG**(C)GGCCACTGCCTGATTTCAAAC**TAGTGAAGCCACAGATGTA**GTTTGAAATCAGGCAGTGGCCA**TGCCTACTGCCTCGGA |
| 2 | ACCUCUUUCAGUCCUGUUUGAA | | TGCTGTTGACAGTGAGCG**CCCTCTTTCAGTCCTGTTTGAA**TAGTGAAGCCACAGATGTA**TTCAAACAGGACTGAAAGAGGT**TGCCTACTGCCTCGGA |

**Supplemental Table 2.** Oligonucleotides primers used in this study

| **Primers** | **Purpose** | **Sequence 5’-3’** |
| --- | --- | --- |
| miR30-F | Knockdown of OCT-1 | CAGAAGGCTCGAGAAGGTATATTGCTGTTGACAGTGAGCG |
| miR30-R | Knockdown of OCT-1 | CTAAAGTAGCCCCTTGAATTCCGAGGCAGTAGGCA |
| hOCT-1-F | hOCT-1-EYFP over expression | AAATCAGATCTCGAGGCCGCCACCATGCCCACCGTGGATGAC |
| hOCT-1-R2 | hOCT-1-EYFP over expression | ACCGTCGACTGCAGAATTCGGGTGCCCGAGGGTTCAGAGGTTTGG |
| Sequencing | hOCT-1-EYFP construct sequencing | CAAATGGGCGGTAGGCGTTG |
| hOCT-1-EYFP_D474C-F | hOCT-1-EYFP site-directed mutagenesis | GTGTTCCTCCCTGTGTTGCATAGGTGGGATAATCA |
| hOCT-1-EYFP_D474C-R | hOCT-1-EYFP site-directed mutagenesis | TTATCCCACCTATGCAACACAGGGAGGAACACACC |

**FIGURE LEGENDS TO SUPPLEMENTAL DATA**

**Figure S1**. Epifluorescent microscopy showing DNR uptake into the nucleus of the ovarian cancer cell line OV866(2).

**Figure S2.** Epifluorescent microscopy showing DNR uptake under different conditions in the ovarian cancer cell line OV866(2).

**Figure S3.** Western blot analysis showing the expression level of OCT1 in various cancer cell lines.

**Figure S4.** Plasma membrane localization of native OCT1-EYFP and its variant OCT1-D474C-EYFP.

**Figure S5**. RT-PCR analysis showing the expression level of CT2 (panel A) and OCTN2 (panel B) in various leukemia cell lines and when cells were untreated and treated with DNR.

**The human organic cation transporter OCT1 mediates high affinity uptake of the anticancer drug daunorubicin**

Emil Andreev, Nicolas Brosseau, Euridice Carmona, Anne-Marie Mes-Masson and Dindial Ramotar


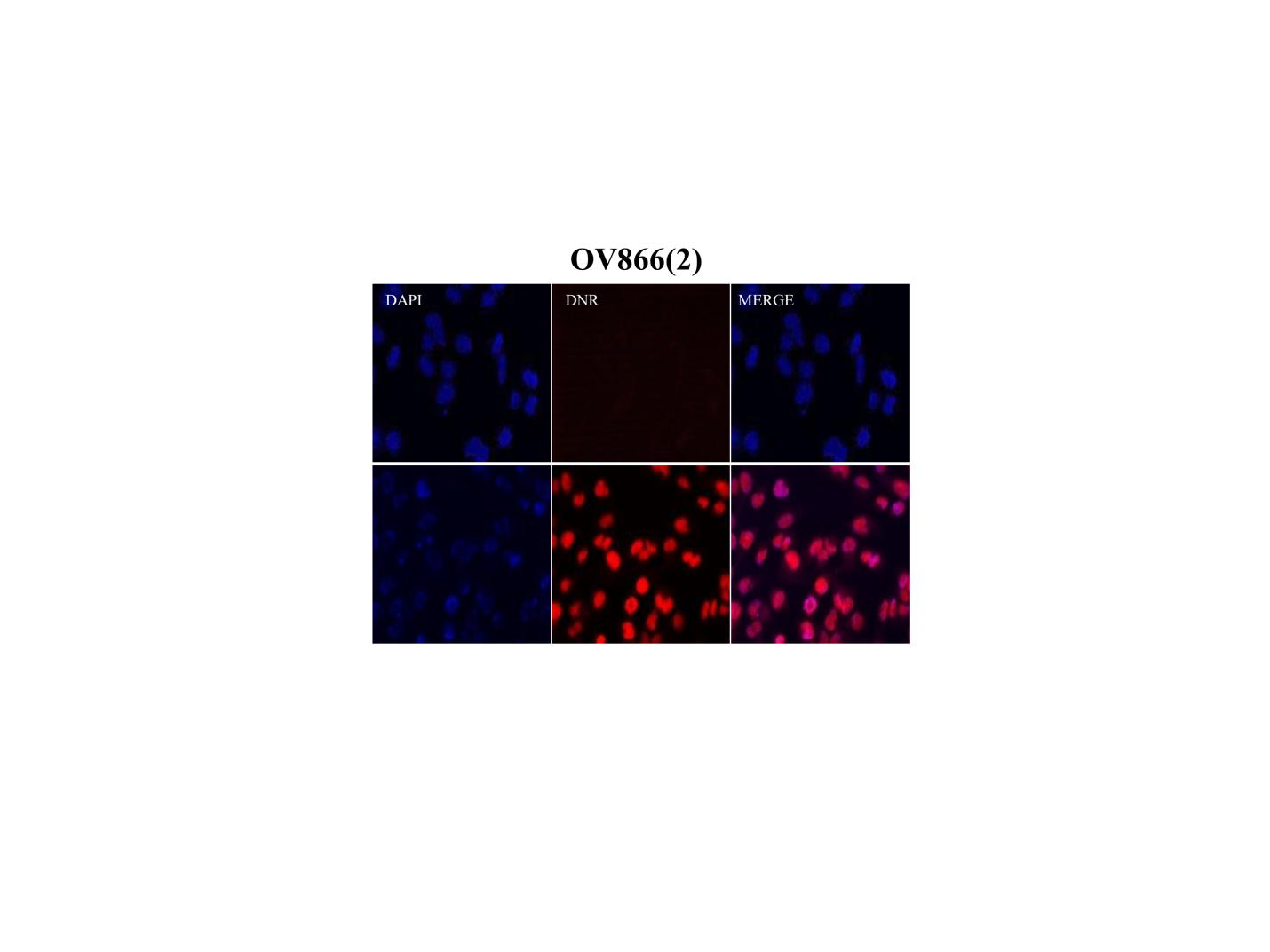


Supplemental data Fig. S1. **Epifluorescent microscopy showing DNR uptake into the nucleus**

**of the ovarian cancer cell line OV866(2)**


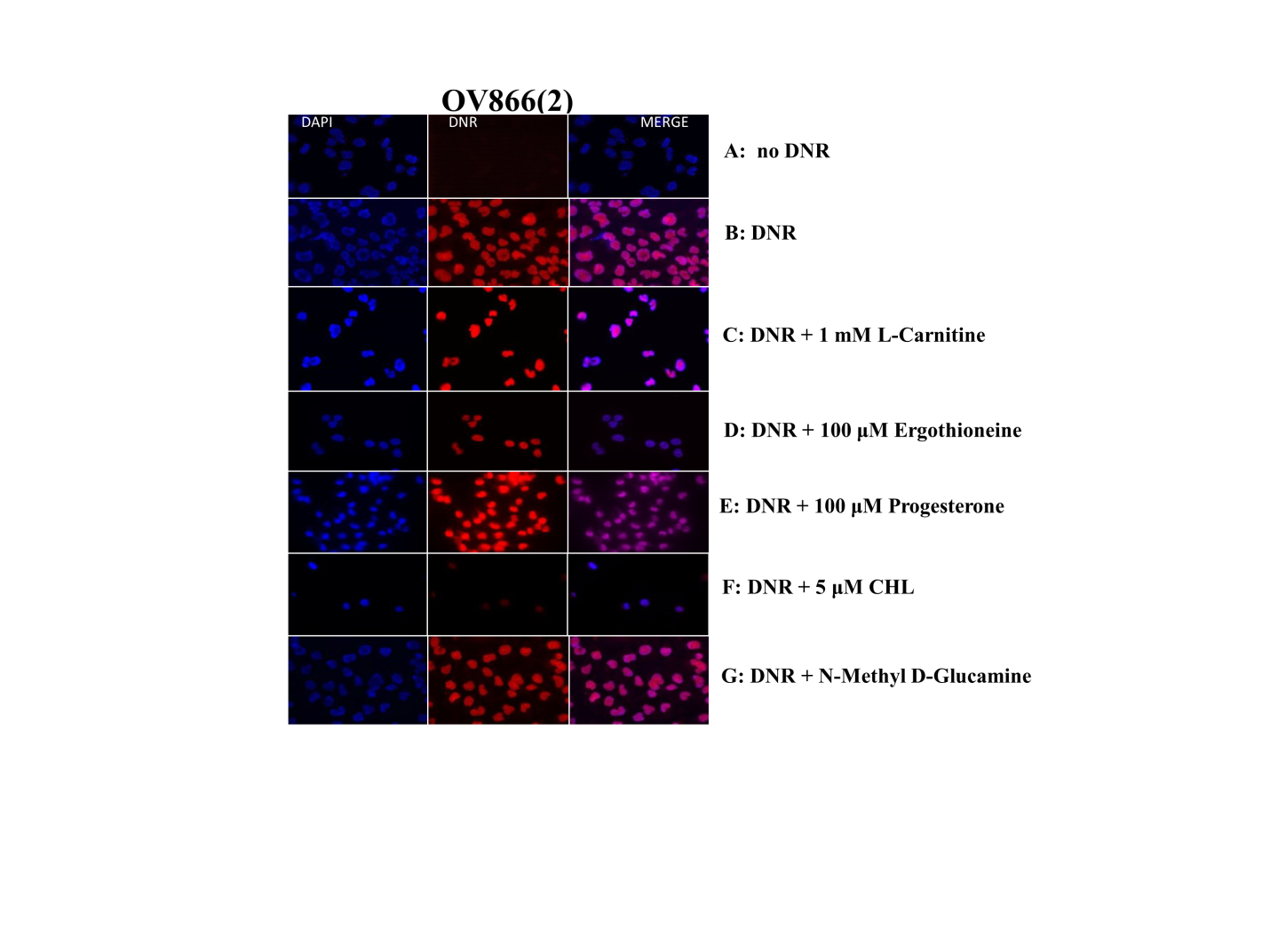


Supplemental data Fig. S2. **Epifluorescent microscopy showing DNR uptake under**

**different conditions in the ovarian cancer cell line OV866(2).**


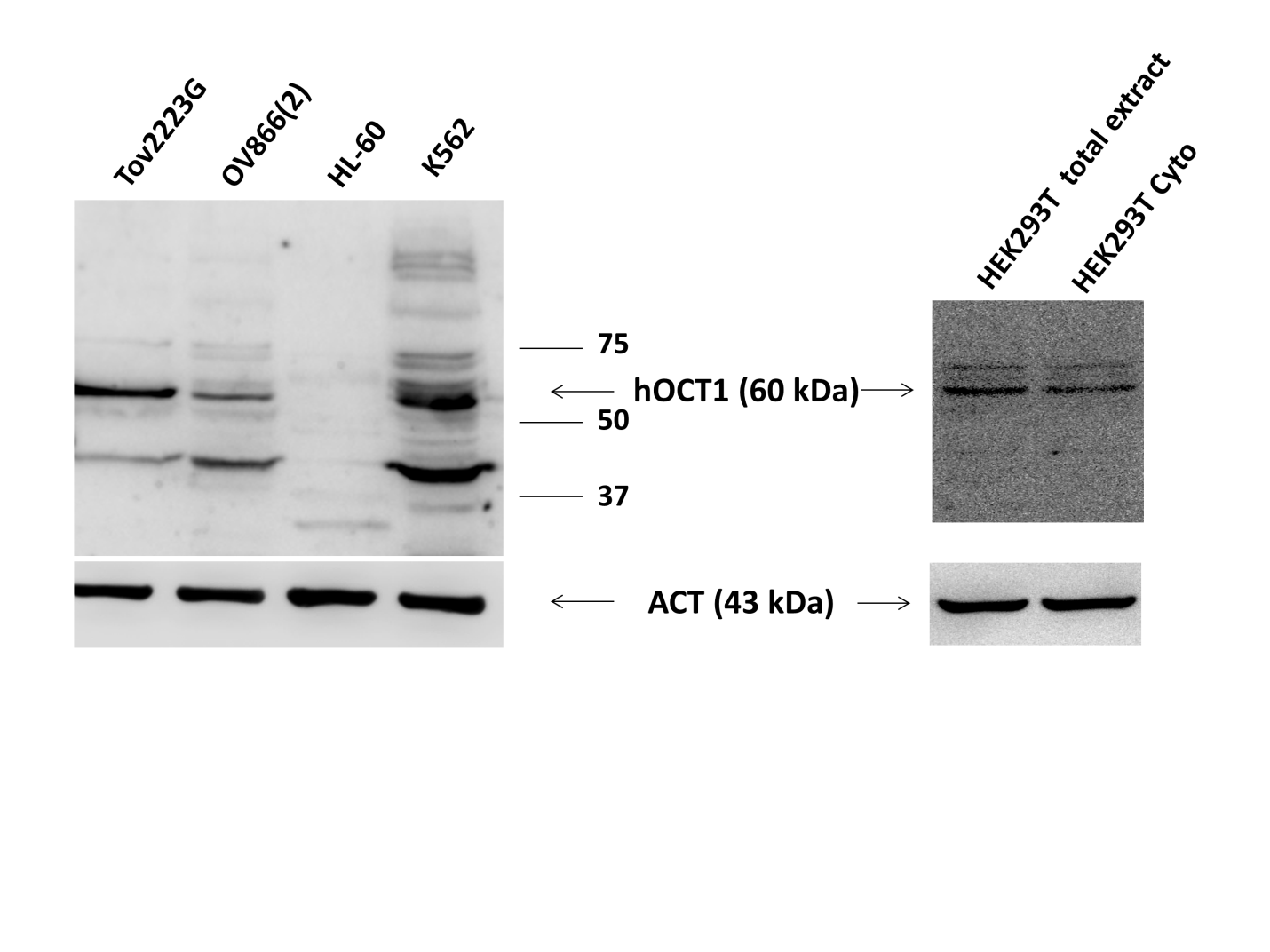


Supplemental data Fig. S3. **Western blot analysis showing the expression level of OCT1**

**in various cancer cell lines**.


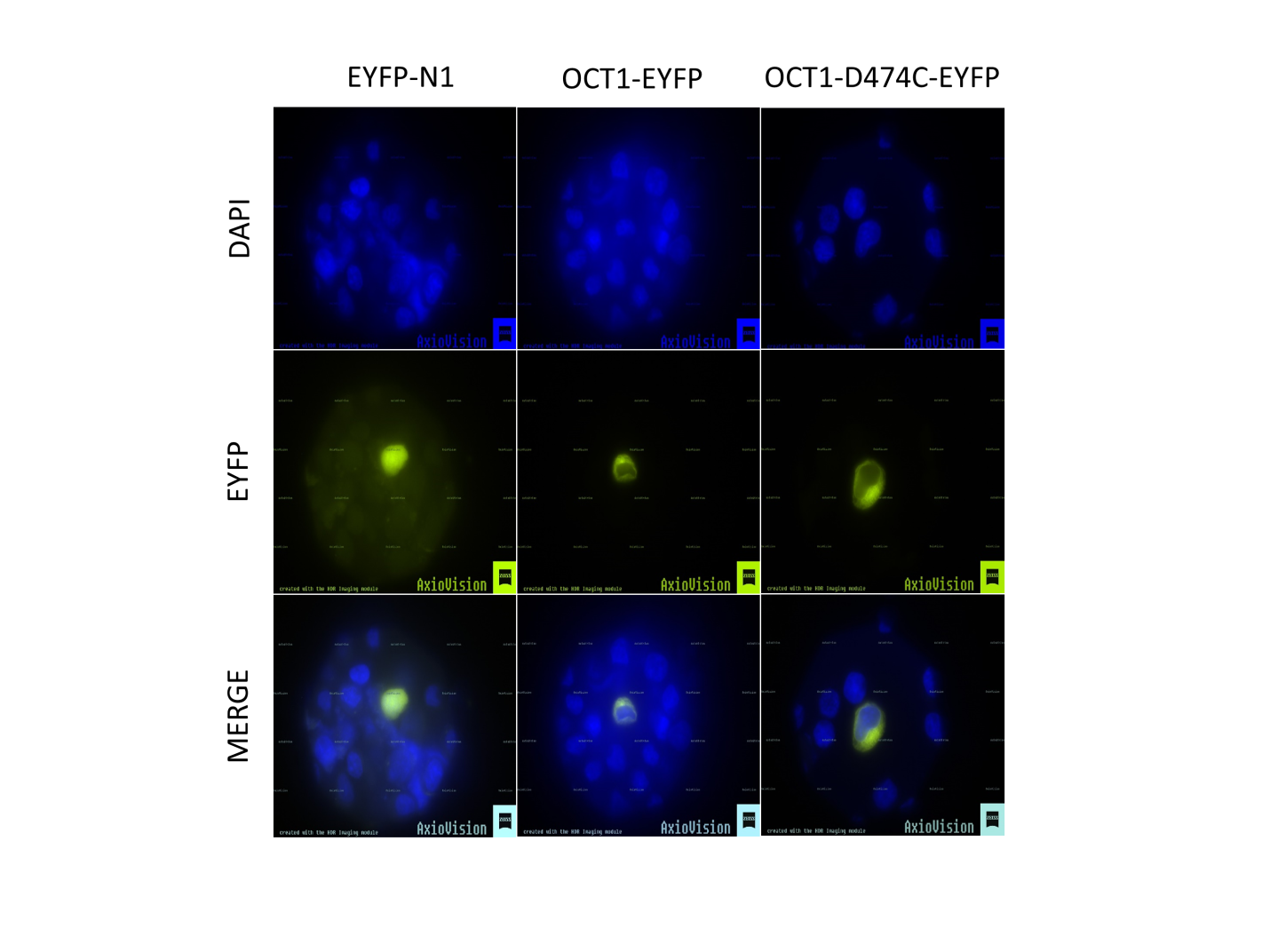


Supplemental data Fig. S4. **Plasma membrane localization of native OCT1-EYFP and its variant**

**OCT1-D474C-EYFP.**


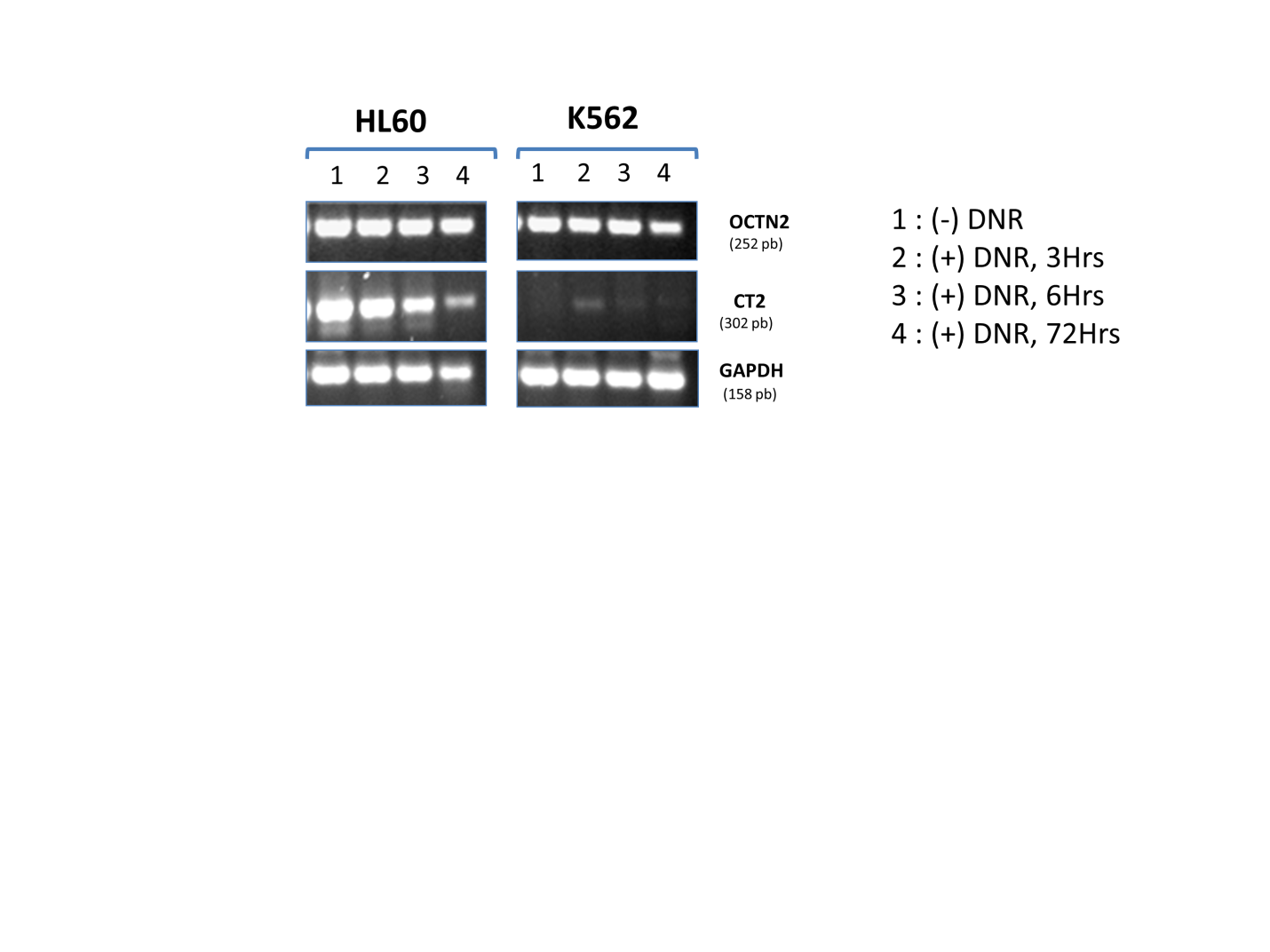


Supplemental data Fig. S5. **RT-PCR analysis showing the expression level of CT2 (panel A)**

**and OCTN2 (panel B) in various leukemia cell lines and when cells were untreated and**

**treated with DNR.**
